# Animal Activities of the Key Herbivore Plateau Pika (*Ochotona curzoniae*) on the Qinghai-Tibetan Plateau Affect Grassland Microbial Networks and Ecosystem Functions

**DOI:** 10.3389/fmicb.2022.950811

**Published:** 2022-07-06

**Authors:** Jiawei Yang, Sijie Wang, Wanghong Su, Qiaoling Yu, Xiaochen Wang, Qian Han, Yuting Zheng, Jiapeng Qu, Xiangzhen Li, Huan Li

**Affiliations:** ^1^School of Public Health, Lanzhou University, Lanzhou, China; ^2^Changsha Central South Forestry Survey Planning and Design Co., Ltd., Changsha, China; ^3^Qinghai Provincial Key Laboratory of Restoration Ecology for Cold Region, Northwest Institute of Plateau Biology, Chinese Academy of Sciences, Xining, China; ^4^Key Laboratory of Adaptation and Evolution of Plateau Biota, Northwest Institute of Plateau Biology, Chinese Academy of Sciences, Xining, China; ^5^Key Laboratory of Environmental and Applied Microbiology, Environmental Microbiology Key Laboratory of Sichuan Province, Chengdu Institute of Biology, Chinese Academy of Sciences, Chengdu, China; ^6^State Key Laboratory of Grassland Agro-Ecosystems, Center for Grassland Microbiome, College of Pastoral Agriculture Science and Technology, Lanzhou University, Lanzhou, China

**Keywords:** microbial network, plateau pika, grazing, disturbance, ecosystem

## Abstract

Plateau pikas (*Ochotona curzoniae*) are high-altitude model animals and famous “ecosystem engineers” on the Qinghai-Tibet Plateau. Pika activities may accelerate the degradation of alpine meadows. Nevertheless, little is known about the responses of bacterial, fungal, and archaeal communities, and ecosystem multifunctionality to pika perturbations. To address this question, we studied the impacts of only pika disturbance and combined disturbance (pika disturbance and grazing) on ecological networks of soil microbial communities and ecosystem multifunctionality. Our results demonstrated that Proteobacteria, Ascomycota, and Crenarchaeota were dominant in bacteria, fungi, and archaea, respectively. Bacteria, fungi, and archaea were all influenced by the combined disturbance of grazing and pika. Most fungal communities became convergent, while bacterial and archaeal communities became differentiated during the succession of surface types. In particular, the bacterial and fungal networks were less stable than archaeal networks. In response to the interference, cross-domain cooperation between bacterial and fungal communities increased, while competitive interactions between bacterial and archaeal communities increased. Pika disturbance at high intensity significantly reduced the ecosystem multifunctionality. However, the mixed effects of grazing and pika weakened such influences. This study revealed how pika activities affected microbial networks and ecosystem multifunctionality. These results provide insights to designing reasonable ecological management strategies for alpine grassland ecosystems.

## Introduction

The Qinghai-Tibet Plateau (QTP) is the highest plateau (average 4,000 m above sea level) on earth, known as “the roof of the world.” There are various types of grasslands on the Qinghai Tibet Plateau, like alpine meadow, alpine grassland, alpine shrub, and alpine desert. Its alpine meadow area reaches 1.39 km × 10^6^ km, occupying nearly half (44%) of the grassland area of China (Tan et al., 2010). It provides many important ecosystem services, including protecting biodiversity, regulating regional climate, and preventing soil erosion (Tilman et al., 1996; Yan et al., 2003; Harper et al., 2005). In recent years, grassland degradation on the QTP causes serious social and ecological problems (Gao et al., 2006; Harris, 2010). Overgrazing, permafrost ablation, and disturbance of small herbivorous animals accelerate the degradation rate of alpine meadows (Zhou et al., 2005; Yang et al., 2010; Zhang et al., 2016). Among these factors, the disturbance of small herbivores are considered to be important factors for grassland degradation on the QTP because of their high population density and burrowing behavior (Shang and Long, 2007; Li et al., 2013).

Plateau pika (*Ochotona curzoniae*), as a key species, is a small herbivorous mammal widely distributed on the QTP. It is also considered an important high-altitude model animal and “ecosystem engineer” (Smith and Foggin, 1999). The foraging behavior of plateau pika can affect the aboveground vegetation (Sun et al., 2015). More importantly, they can change the microenvironment and promote the material circulation of the ecosystem by burrowing behavior (Jones et al., 1994; Singleton et al., 1999). Pika disturbance leads to the succession of grassland plant communities, gradually forming a “grassland – new mound – old mound – bald patch” surface type (Ma et al., 2018). Concretely, the burrowing behavior of pika forces part of the grassland to produce a new mound, where the soil is dug out of the cave and becomes relatively loose. When the pika burrows are in excessive numbers, some of them are discarded, thereby forming the old mounds. The old mound soil is compacted tightly due to animal activities such as stepping, forming a bald patch (i.e., bare land) eventually. Higher burrow densities of pika can increase soil carbon and nitrogen contents (Sun et al., 2015), while linearly reducing net ecosystem CO_2_ exchange and above-ground biomass (Liu et al., 2013). To date, most studies primarily focus on the impact of pika interference on soil physicochemical and vegetation properties (Liu et al., 2013; Sun et al., 2015; Wu et al., 2015), but little attention is paid to soil microbial communities and ecosystem multifunctionality (EMF).

Soil microorganisms provide many important ecosystem functions, for example, productivity, nutrient cycling, organic matter degradation, and plant growth (Van Der Heijden et al., 2008; Wall et al., 2012). Soil microbiomes are influenced by climate, topography, soil conditions, animal activities, vegetation, and even human disturbances (Delgado-Baquerizo et al., 2014; Eldridge et al., 2016; Wei et al., 2019; Tajik et al., 2020). Previous research has shown that soil microbial diversity, as an important component of subsurface nutrient taxa, drives EMF (Wagg et al., 2014). The positive relationship between plant diversity and EMF is shown to be indirectly driven by soil biodiversity (Delgado-Baquerizo et al., 2020). Many researchers emphasize the impact of the enormous biodiversity underground on the ecosystem multifunctionality (Bardgett and van der Putten, 2014; Delgado-Baquerizo et al., 2016, 2020). For example, the biodiversity of bacteria, fungi, and protozoa play irreplaceable roles in the operation of ecosystem function under a low functional threshold (Delgado-Baquerizo et al., 2020). There may be a division of metabolic labor between microorganisms that leads to the complementarity of different taxa, suggesting that such unseen interactions may have broader implications for soil ecological function (Wagg et al., 2019). Nevertheless, there are few studies on whether the ecosystem multifunctionality is affected by the interaction of microbial communities, or by pika disturbance on the QTP.

Ecological network analysis is a widely used approach to explain community interactions (Faust et al., 2012; Deng et al., 2016; Li et al., 2017). For instance, soil-foraging animals (e.g., bilbies and bettongs) are recognized to change the networks of bacterial and fungal communities in desert shrubland (Eldridge et al., 2015). Generally, microbial networks consist of nodes and edges. Nodes are generally species or other taxa, and edges are connections (or associations) between nodes. The cooperative relationship between microbial species is represented by positive links between nodes, while negative links indicate mutual competition (Faust et al., 2012). Moreover, the topological features of the network can also reveal valuable biological information (Ma et al., 2016). For example, network degree and density can reflect the complexity of interspecific interactions (Wu et al., 2016). Modularity values indicate functional units or niches in microbial communities (Luo et al., 2006; Eiler et al., 2012). Therefore, network analysis can be used to reveal the changes in microbial interactions under disturbance in our study.

Here, a field control experiment and the next-generation sequencing were used to systematically study the impact of grazing and pika disturbance on soil microbial networks and ecosystem multifunctionality (EMF). Based on previous research, pika perturbation increased carbon and nitrogen and decreased plant diversity (Sun et al., 2015; Wu et al., 2015), and might simplify the microbial ecological networks and soil functions. The main goals were to reveal (1) the influence of pika interference on microbial communities and EMF; (2) the changes in co-occurrence networks of soil microbiomes under disturbance; (3) the relationships among microbial communities, co-occurrence networks, and EMF.

## Materials and Methods

### Experimental Design and Sample Collection

The field experiment was conducted in a typical alpine meadow of Qinghai-Tibet Plateau from June 20 to 26, 2016. The meadow is located near Haibei Alpine Meadow Ecosystem Research Station in Menyuan County, 155 km north of Xining, Qinghai Province, China (37°37′N, 101°12′E). Six blocks were selected, of which two blocks with combined disturbance (pika and livestock, PL), two blocks with only pika disturbance but no grazing (P), and two blocks without pika disturbance and grazing (natural meadow, C). Based on the walked transect method (Li et al., 2016), the pika population densities (individuals/hm^2^) in four blocks (P and PL) were 38.5–56 and were considered to be high population densities (Li et al., 2016). Five sites were taken in each block for sampling at 0–10 cm depth. Each site was spatially separated by more than 50 m. In the P and PL blocks, each site was divided into five quadrats (10 cm × 10 cm), including inside the mound (I), new mound (N), old mound (O), bare land (B), and grassland (G). In the C block, each site only sampled natural meadow soil. Before soil sampling, the total coverage of vegetation within each quadrat was investigated. The above-ground plants were clipped and contained in the envelopes. Soil samples were collected into a 50 mL sterile tube following the five-spot-sampling method and kept in a box with ice packs. The samples were then transferred to our laboratory and frozen at −20°C. At last, a total of 110 soil samples were collected.

### Physicochemical Properties and Vegetation Biomass Analysis

Soil physicochemical properties were measured based on routine soil analysis methods, including pH, conductivity (CON), ammonium nitrogen (NH_4_-N), nitrate-nitrogen (NO_3_-N), soil organic carbon (SOC), and total nitrogen (TN). The pH and CON were estimated by a bench pH water quality analyzer (AZ86505, Shenzhen Lemaiyi Electronics co., Ltd.). Fresh soil samples (10 g each) were sieved through 20 mesh stainless steel. Then NH_4_-N and NO_3_-N were determined according to the previous study (Feng et al., 2021). The Walkley and Black method was used to detect SOC (Walkley, 1935). The TN values were determined by the Kjeldahl method (Bremner, 1965). The total plant coverage was estimated based on the quadrat survey (Ji et al., 2009). The main plant species in the meadow are *Kobresia humilis*, *Festuca ovina*, *Potentilla bifurca*, *Potentilla anserina*, *Polygonum viviparum*, etc. For plant biomass measurement, because the area of each mound is approximately 0.02–0.05 m^2^, all plant material was collected from a 0.01 m^2^ quadrat, oven-dried and weighted (Ji et al., 2009).

### Soil DNA Extraction and Miseq Sequencing

Our previous study (Yang et al., 2021) described the details of DNA extraction, PCR amplification, and gel extraction. Primer pairs 515F and 909R (5′-GTG CCA GCM GCC GCG G-3′ and 5′-CCC CGY CAA TTC MTT TRA GT-3′) were used to amplify bacteria (Tamaki et al., 2011). Primer pairs 344F and 806R (5′-ACG GGG YGC AGC AGG CGC GA-3′ and 5′-GGA CTA CVS GGG TAT CTA AT-3′) were used to amplify archaea (Takahashi et al., 2014). Primer ITS4 and ITS7 (5′-TCC TCC GCT TAT TGA TAT GC-3′ and 5′-GTG ART CAT CGA RTC TTT G-3′) were used to amplify fungal ITS genes (Bokulich and Mills, 2013). The PCR products were sequenced on the Illumina MiSeq sequencer (Illumina, San Diego, CA, United States).

### Bioinformatics Analysis

QIIME pipeline was used to perform the original sequences for bioinformatics analysis (Li et al., 2019). The primer sequences were distinguished by unique barcode and then combined with FLASH v1.2.8 (Mago and Salzberg, 2011). The combined sequences were processed to remove low-quality sequences and chimeras (Edgar et al., 2011), and the operational taxonomic units (OTUs) were generated with a sequence similarity threshold of 97% (Edgar, 2010). Then the OTU tables removed chloroplast were re-sampled to the same sequence based on the “daisyhopper” script (Gilbert et al., 2009). Each sample obtained 12,135 bacterial sequences, 9,739 fungal sequences, and 13,550 archaeal sequences. Then species classifications were carried out based on the ribosomal database project (RDP) (Wang et al., 2007). The alpha diversity calculated in QIIME included Chao 1, Observed OTUs, Shannon and Simpson diversity (Li et al., 2017). The weighted and unweighted UniFrac distance matrices were calculated to evaluate the beta diversity, and the scores were drawn as the principal component analysis (PCA) (Hamady et al., 2010). The community dissimilarity was generated according to the Bray-Curtis distance matrices, in which the community similarity was obtained by subtracting the community dissimilarity from 1 (Yu et al., 2022).

### Co-occurrence Networks Analysis

The co-occurrence network was constructed with the dominant OTUs (relative abundance > 0.01%). The correlation coefficient between OTUs (*r*) was analyzed by the methods of Spearman rank correlations based on the R 4.1.0. The cross-domain networks were constructed by deleting the internal correlations of bacteria, fungi, and archaea. The networks were visualized and analyzed by Gephi v0.9.2 and the R “psych” package (| *r*| > 0.7, *P* < 0.05) (Bastian et al., 2009). Next, the topological features of each sub-network were calculated at the network level (node, edge, average degree, modularity, diameter, density, positive, and negative correlation proportions), the node level (average clustering coefficient), and the edge level (average path length) (Li et al., 2019).

### Ecosystem Multifunctionality

The ecosystem multifunctionality in our study was estimated by these variables related to the support and regulation of ecosystem services: (1) total coverage of vegetation, (2) dry weight of vegetation, (3) wet weight of vegetation, (4) ammonium nitrogen of soil, (5) nitrate-nitrogen of soil, (6) soil organic carbon, (7) total nitrogen of soil (Suen et al., 2010). The indices of EMF were obtained by averaging the *Z*-scores (the measured value minus the mean divided by the standard deviation) for the measured variables (Maestre et al., 2012; Byrnes et al., 2014).

### Statistical Analysis

Soil environmental factors and EMF in each group were statistically compared by one-way analysis of variance (one-way ANOVA) with Tukey’s *post hoc* test based on SPSS v26.0 (SPSS Inc., Chicago, IL, United States). The effects of surface type (i.e., grassland, new mound, old mound, bare land, and inside the mound) and disturbance type (i.e., combined and only disturbances) on environmental factors, EMF, main phyla, and genera of microbial communities were calculated by two-way analysis of variance (two-way ANOVA). The Linear discriminant analysis effect size (LEfSe) was used to demonstrate genera enriched in different habitats^1^ (Guo et al., 2019). The enrichment ratios of OTUs were calculated by analysis of variance (ANOVA). The difference in alpha diversity in each group was analyzed by the Mann–Whitney *U*-Test. The influence of only pika and mixed disturbance on beta diversity was determined by permutational multivariate analysis of variance (PERMANOVA) (McArdle and Anderson, 2001). The effects of mixed disturbance, only pika disturbance, and environmental factors on soil microbial community structures were calculated by PERMANOVA and the Mantel test. Additionally, the multiple regression matrix (MRM) was used to detect the significant contributions of those factors to microbial community structures (Deng et al., 2016). Heatmap was constructed by the R “pheatmap” package to show relationships among environmental factors, microbial diversity, and major genera. The calculation and display of linear regression were generated by Origin v8.5 (OriginLab, Northampton, MA, United States).

## Results

### The Changes in Plant Biomass and Soil Physicochemical Properties

Plant biomass and soil physicochemical properties were altered by surface type and disturbance type (Supplementary Figure 1 and Supplementary Table 1). For example, the total coverage of vegetation in the grassland (PG and PLG) increased compared with the natural meadow (C), while it decreased significantly in the new mound (PN and PLN) (*P* < 0.05; Supplementary Figure 1A). The changing trends of wet and dry weights of vegetation were consistent with the total coverage (Supplementary Figures 1B,C). Soil pH was significantly increased and remained alkaline compared to the natural meadow (*P* < 0.05; Supplementary Figure 1D), and the CON was also constantly increased (Supplementary Figure 1E). The NO_3_-N content of the new mound was higher than that of the grassland (*P* < 0.05; Supplementary Figure 1F). Compared with the natural meadow, NH_4_-N in the PG and PLG groups increased significantly (*P* < 0.05; Supplementary Figure 1G). However, the concentration of NH_4_-N showed a tendency downward from the grassland to bare land. The changes in TN and SOC were similar to those of NH_4_-N (Supplementary Figures 1H,I). The NH_4_-N, TN, and SOC were higher under mixed disturbance than under only pika disturbance. Collectively, all environmental factors were influenced by disturbance type significantly, while surface type significantly affected vegetation biomass, NO_3_-N, pH, and CON (Two-way ANOVA, *P* < 0.05; Supplementary Table 1).

### Effect of Disturbance on Soil Microbiomes

A total of 7,816,920 high-quality bacterial reads (Mean: 71,063; Max: 128,794; Min: 21,965; *SD*: 16,361), 11,123,473 high-quality fungal reads (Mean: 101,122; Max: 226,496; Min: 17,804; *SD*: 38,288), and 6,398,926 high-quality archaeal reads (Mean: 58,172; Max: 101,532; Min: 14,813; *SD*: 11,632) were obtained from 110 soil samples.

**FIGURE 1:**
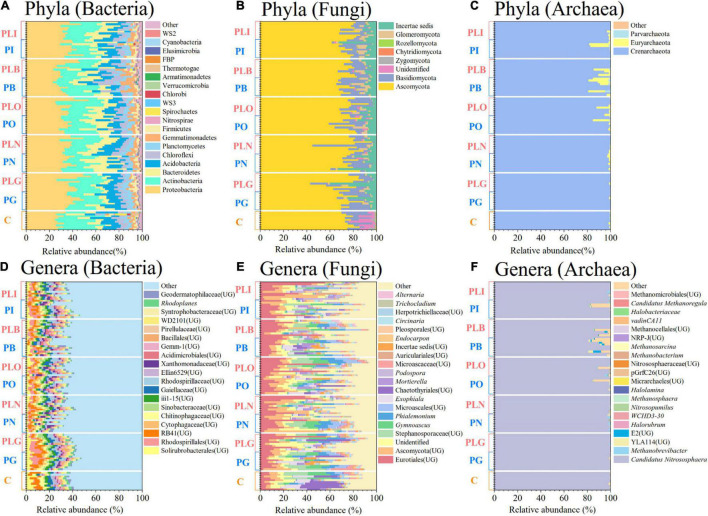
The compositions of soil bacterial **(A,D)**, fungal **(B,E)**, and archaeal **(C,F)** communities at the phylum (top) and genus (bottom) level. Only dominant genera with the top 20 mean relative abundance were shown. UG, unidentified genus; C, natural grass; P, pika disturbance; PL, grazing and pika disturbance; G, grassland; N, new mound; O, old mound; B, bare land; I, inside the mound.

The main bacterial phyla comprised Proteobacteria (average relative abundance: 35.6%), Actinobacteria (21.8%), Bacteroidetes (11.6%), Acidobacteria (10.3%), and Chloroflexi (5.8%) (Figure 1A). The relative abundances of Bacteroidetes and Chloroflexi were significantly affected by surface type, and Proteobacteria was influenced by both disturbance and surface types (Two-way ANOVA, *P* < 0.05; Supplementary Table 2). Solirubrobacterales (UG, unidentified genus) (3.3%), Rhodospirillales (UG) (3.0%), and RB41 (UG) (3.0%) were the major bacterial genera (Figure 1D). Solirubrobacterales (UG) was enriched in the grassland (LEfSe, *P* < 0.05; Supplementary Figure 2A). Besides, many bacterial genera [e.g., Rhodospirillales (UG), RB41 (UG), Chitinophagaceae (UG)] were significantly influenced by combined disturbance of grazing and pika (Two-way ANOVA, *P* < 0.05; Supplementary Table 3).

Soil dominate fungal phyla were Ascomycota (75.9%), Basidiomycota (12.4%), Incertae sedis (7.2%), and Glomeromycota (2.5%) (Figure 1B). Incertae sedis, Zygomycota, and Chytridiomycota were significantly affected by disturbance type, and Glomeromycota was impacted by surface type (Two-way ANOVA, *P* < 0.05; Supplementary Table 2). Eurotiales (UG) (15.5%), Ascomycota (UG) (9.0%), and Stephanosporaceae (UG) (5.3%) were the main fungal genera (Figure 1E). Ascomycota (UG) was significantly abundant in the new mound and old mound (LEfSe, *P* < 0.05; Supplementary Figure 2B). Eurotiales (UG) was significantly affected by mixed disturbance, and only pika disturbance had an impact on Ascomycota (UG) (Two-way ANOVA, *P* < 0.05; Supplementary Table 3).

The soil archaeal phyla mainly consisted of Crenarchaeota (98.0%) (Figure 1C). The surface type had a significant effect on archaeal phyla (Two-way ANOVA, *P* < 0.05; Supplementary Table 2). Candidatus *Nitrososphaera* (97.9%) was the most abundant genera of archaea and abundant in the grassland (Figure 1F and Supplementary Figure 2C). Candidatus *Nitrososphaera*, *Methanobrevibacter*, E2 (UG) were mainly affected by surface type, while not influenced by disturbance type (Two-way ANOVA; Supplementary Table 3).

**FIGURE 2:**
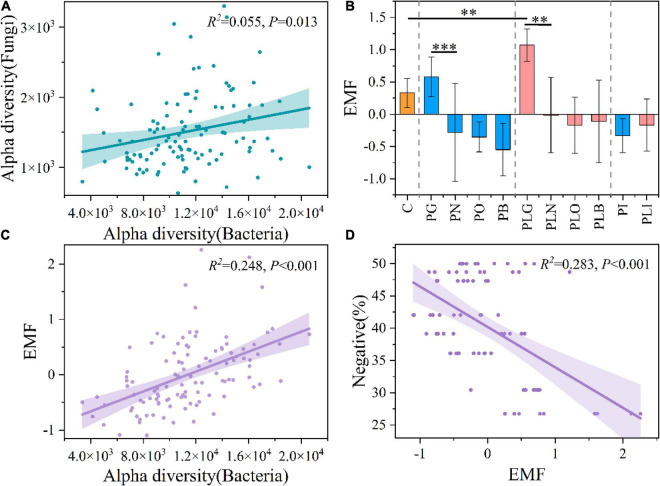
The linear regression between bacterial and fungal α diversity **(A)**. The ecosystem multifunctionality (EMF) in each group **(B)**. The linear regression between EMF and bacterial α diversity **(C)**, and between EMF and negative proportion of networks in Figure 7 **(D)**. The area above and below the fitting line represents the 95% confidence interval. C, natural grass; P, pika disturbance; PL, grazing and pika disturbance; G, grassland; N, new mound; O, old mound; B, bare land; I, inside the mound. Significant indicator by ^**^P < 0.01 and ^***^P < 0.001.

In addition, Chao 1 index of bacteria was much higher than that of fungi and archaea (Supplementary Figure 3 and Supplementary Table 4). Both the only pika and combined disturbance significantly decreased soil alpha diversity of bacteria (Mann–Whitney *U*-Test, *P* < 0.05; Supplementary Table 4). The fungal diversity increased under the only pika disturbance, while decreased under the mixed disturbance (Supplementary Figure 3 and Supplementary Table 4). Different from the fungi and bacteria, soil archaeal diversity increased under the joint disturbance (in the old mound, especially) (Supplementary Figure 3). Interestingly, the bacterial α diversity (Chao 1) had a significant positive correlation with the fungal diversity (*R*^2^ = 0.055, *P* < 0.05; Figure 2A). But there was no significant relationship between the diversity of fungi and archaea, archaea, and bacteria (*P* > 0.05; Supplementary Figures 6A,B). Pika disturbance had significant effects on the β diversity of bacteria and archaea (in grassland and new mounds, particularly), while not on fungi (Supplementary Figure 4 and Supplementary Table 5).

**FIGURE 3:**
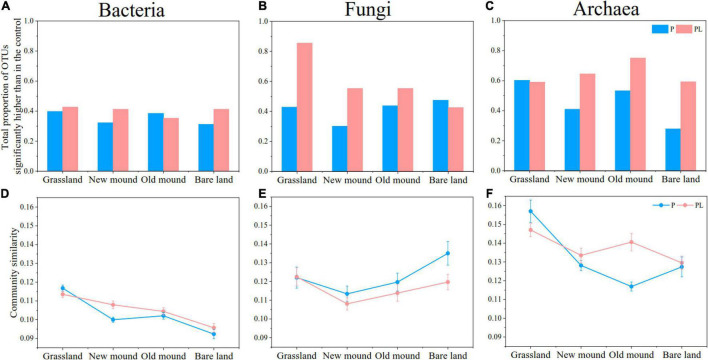
The proportions of total bacterial **(A)**, fungal **(B)**, and archaeal **(C)** OTUs significantly enriched in each group compared with the natural grassland by Analysis of Variance (ANOVA) (all *P* < 0.05). The community similarity of bacterial **(D)**, fungal **(E)**, and archaeal **(F)** communities in each group based on Bray–Curtis distance metrics. P (blue), pika disturbance; PL (pink), grazing and pika disturbance.

The enrichment ratio of fungi and archaea OTUs was much higher in response to mixed and only pika disturbance than that of bacterial OTUs (Figures 3A–C). The enrichment ratio of archaea remained almost above 50%, while the fungal enrichment decreased continuously from grassland to bare land (especially in PL groups, 85.46–42.53%) (Figures 3B,C). The mixed disturbance increased the enrichment proportion of the microbial communities, especially that of archaea (Figure 3C). Based on Bray-Curtis dissimilarity, only pika disturbance reduced the similarity of bacterial and archaeal communities and increased the similarity of fungal communities (Figures 3D–F). However, the change range of the combined groups was not as large as that of the only pika groups. In other words, pika disturbance shifted bacterial and archaeal communities toward difference and fungal communities toward similarity, while mixed disturbance was able to retard this change to some extent.

### Microbial Communities and Their Driving Factors

**FIGURE 4:**
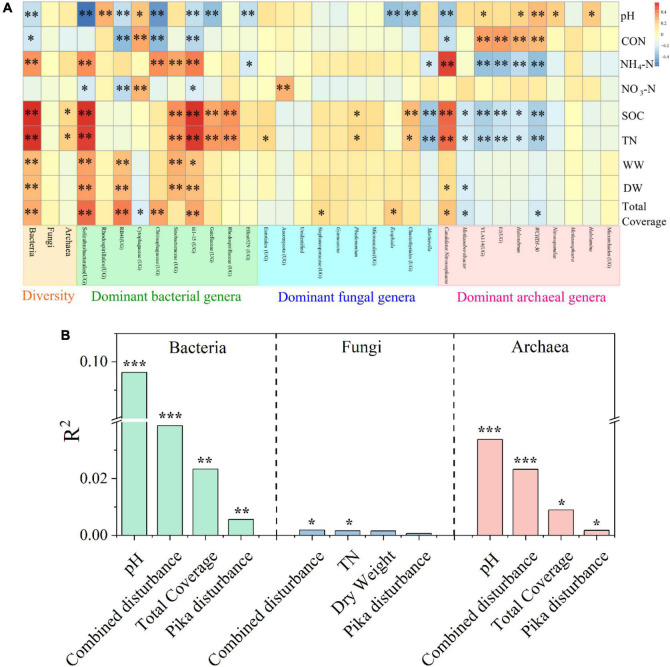
Heatmap showing the correlation coefficients between environmental factors and the α diversity and main genera (the top ten genera of relative abundance) of bacteria, fungi, and archaea based on Spearman’s rank correlation test **(A)**. Multiple regression on distance matrices (MRM) was used to estimate the relative proportions of variance explained by driving factors (top 4) in the communities **(B)**. CON, conductivity; NH_4_-N, ammonium nitrogen; NO_3_-N, nitrate-nitrogen; SOC, soil organic carbon; TN, total nitrogen; WW, wet weight; DW, dry weight; UG, unidentified genus. Significant indicator by *P < 0.05, ^**^P < 0.01, and ^***^P < 0.001.

The bacterial diversity was significantly correlated with all environmental factors except NO_3_-N, archaeal diversity was related to SOC and TN, but there was no significant correlation between fungal diversity and all environmental factors (Figure 4A). Environmental factors had significant correlations with many dominant bacterial and archaeal genera, whereas less with fungal genera (Figure 4A). In addition, pH was the most driving factor for bacterial (*R*^2^ = 0.096, *P* < 0.001) and archaeal (*R*^2^ = 0.034, *P* < 0.001) communities, while fungal communities were barely driven by any measured factors (except mixed disturbance and TN) (Figure 4B). The community structures of bacteria (*R*^2^ = 0.039, *P* < 0.05), fungi (*R*^2^ = 0.002, *P* < 0.05), and archaea (*R*^2^ = 0.052, *P* < 0.05) were driven by combined disturbance (Supplementary Table 6). Combined disturbance (*R*^2^ = 0.039, *P* < 0.05), only pika disturbance (*R*^2^ = 0.006, *P* < 0.05), and environmental factors (*R*^2^ = 0.024, *P* < 0.05) had more effect on bacteria than on fungi and archaea (Supplementary Table 6). Furthermore, bacterial community and archaea were influenced most by total plant coverage (*R*^2^ = 0.206, *P* < 0.05) and mixed disturbance (*R*^2^ = 0.052, *P* < 0.05), respectively (Supplementary Table 7). The results of the Mantel test were broadly consistent with those of PERMANOVA (Supplementary Table 11).

### Network Succession of Microbial Communities

**FIGURE 5:**
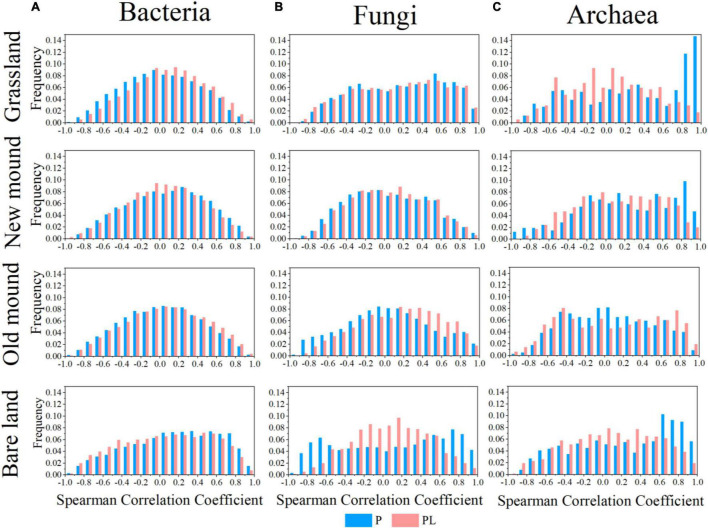
Frequency distributions of all correlations in the bacterial **(A)**, fungal **(B)**, and archaeal **(C)** networks in the grassland, new mound, old mound, and bare land (top to bottom) based on Spearman’s rank correlation test. P (blue), pika disturbance; PL (pink), grazing and pika disturbance.

Taking all correlations into account, we obtained frequency distribution plots of correlation coefficients for bacteria, fungi, and archaea in grassland, new mound, old mound, and bare land (Figure 5). The potential links between OTUs under only pika disturbance were stronger than those under mixed disturbance, especially fungi. The strongly correlated proportions of fungal networks increased obviously during the succession of surface type; however, this change was not evident under the combined disturbance (Figure 5B). This suggested, only pika disturbance increased the network correlation of fungi, but the mixed disturbance delayed this change.

**FIGURE 6:**
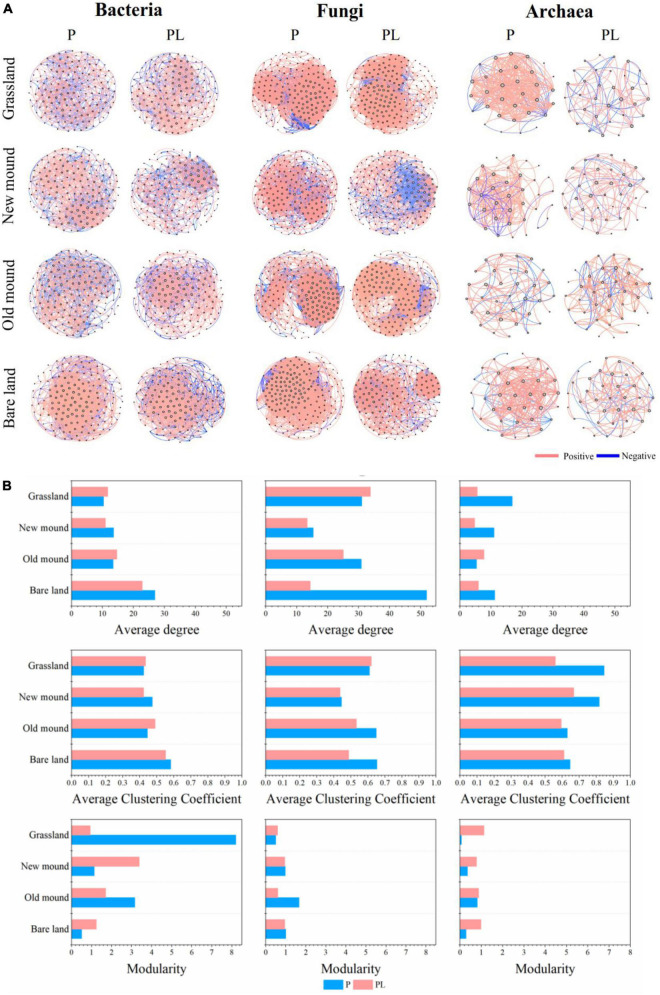
The bacterial, fungal, and archaeal networks **(A)** and properties **(B)** in the grassland, new mound, old mound, and bare land. Nodes represent main OTUs (relative abundance > 0.01%); edges represent significant Spearman correlations (|*r*| > 0.7, *P* < 0.05). P (blue), pika disturbance; PL (pink), grazing and pika disturbance. (For detailed network properties, see Supplementary Table 8).

Then, the networks of bacteria, fungi, and archaea were constructed, respectively, with significant correlations (| *r*| > 0.7, *P* < 0.05) (Figure 6A). Fungal and bacterial communities had larger and more complex co-occurrence networks than archaeal communities. The topological features of bacterial, fungal, and archaeal networks in each group were summarized in Supplementary Table 8. The bacterial networks had a lower average clustering coefficient and density, higher modularity, and a higher proportion of negative correlations than the fungal and archaeal networks (Figure 6B and Supplementary Figure 5A). But the network complexities of the combined groups decreased compared to those of the only pika groups, especially the average degree (Figure 6B). Additionally, the archaeal networks were the simplest, and the topological features varied in fluctuation under disturbance (Figure 6B and Supplementary Figure 5C).

**FIGURE 7:**
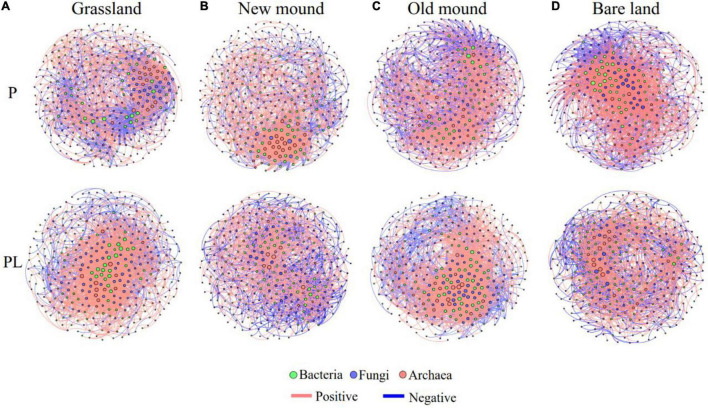
The microbial networks in the grassland **(A)**, new mound **(B)**, old mound **(C)**, and bare land **(D)**. Nodes represent main OTUs (relative abundance > 0.01%) of bacterial (green), fungal (blue), and archaeal (pink) communities; edges represent significant Spearman correlations (| *r*| > 0.7, *P* < 0.05). Pink and dark blue edges indicate positive and negative correlations, respectively. P, pika disturbance; PL, grazing and pika disturbance. (For detailed network properties, see Supplementary Tables 9,10).

Correlations among bacteria, fungi, and archaea were used to construct cross-domain networks (Figure 7). Pika disturbance changed the cross-domain networks among bacteria, fungi, and archaea. The nodes of bacteria and fungi at the center of the network showed changes in different stages of pika disturbance (Figure 7). Besides, the average clustering coefficient of the mixed groups increased from new mound to bare land and was higher than that of the only pika groups (Supplementary Table 9). The bacterial, fungal, and archaeal communities were dominated by positive connections, while the proportion of negative connections increased under combined disturbance (Supplementary Table 9). Specifically, we summarized the connection, positive proportion, and negative proportion among bacteria, fungi, and archaea in the cross-domain networks (Supplementary Table 10). Fungal-bacterial connections accounted for the largest proportion of cross-domain networks. Under the only pika disturbance, the linkages between bacterial and fungal communities increased, and the positive correlation ratio increased from 49.84 to 75.52% (Supplementary Table 10). Under the mixed disturbance, the fungal-bacterial connections decreased gradually. The connections between archaea and bacteria increased significantly, especially the negative correlation ratio (from 12.50 to 39.78%) (Supplementary Table 10).

### The Ecosystem Multifunctionality Under the Only Pika and Mixed Disturbance

The ecosystem multifunctionality (EMF) was influenced by surface and disturbance types (Supplementary Table 1 and Figure 2B). The EMF of the PLG group had a significant increase compared with the native meadow, while the formation of new mounds reduced the EMF to a negative value significantly (ANOVA, *P* < 0.05; Figure 2B). From grassland to bare land, the EMF was continuously decreased. But that in the mixed groups was always higher than that in the only pika groups (Figure 2B). In other words, the EMF was reduced by surface type (*F* = 23.576, *P* < 0.05), and mixed interference delayed this change (Two-way ANOVA; Supplementary Table 1). The least-square linear regression showed that EMF was positively correlated with bacterial alpha diversity (*R*^2^ = 0.248, *P* < 0.05; Figure 2C), but not with fungal and archaeal alpha diversity (Supplementary Figures 6C,D). Moreover, it was negatively correlated with the proportion of negative correlations and positively correlated with the average path length and diameter of cross-domain networks (Figure 2D and Supplementary Figures 6E,F). That is, the decrease in EMF was related to the decrease in bacterial alpha diversity and the topology of microbial cross-domain networks.

## Discussion

This study indicated the significant impacts of only pika disturbance and combined disturbance (including pika disturbance and grazing) on soil microbial communities. Notably, the study concluded that bacterial and fungal networks may be less stable than archaeal networks and increased cross-domain cooperation under the pika disturbance. As expected, pika disturbance at high intensity increased network complexity and reduced ecosystem multifunctionality (EMF). But the combined effects of grazing and pika disturbance delayed these changes. It provides a reference for understanding the response of soil ecosystem multifunctionality and microbial community networks to external perturbations.

### Different Responses to Disturbance With Bacterial, Fungal, and Archaeal Communities

Bacterial, fungal, and archaeal communities differed in the direction of structural change in response to disturbance, which suggested the differential niche selection. Firstly, the significant enrichment ratio of the bacterial community was lower than that of fungi and archaea under the only pika disturbance and combined disturbance interference. Previous studies have shown that bacterial communities are less stable to drought than fungal communities (de Vries et al., 2018), which indicated that many bacterial communities may not adapt to soil moisture changes after plant reduction. However, bacterial communities differentiate under pressure to retain some groups that adapt to interference. Alternatively, it is the structural alteration caused by the invasion of species in excretion from pikas or livestock. For instance, Bacteroidetes are the predominant taxa in the pika stomachs (Li et al., 2017), leading to a positive correlation between Bacteroidetes abundance and soil nutrition. This may be the primary reason for their increased abundance by the effects of pika activities. In addition, in the bare soil environment, the area exposed to ultraviolet radiation increased. The abundance of photoautotrophic microbiomes (e.g., Chloroflexi, Cyanobacteria, and Euryarchaeota) might increase in abundance (Freeman et al., 2009).

Secondly, grassland housed abundant and diverse fungal communities. But the enrichment ratio of fungal communities decreased significantly during the succession of surface types. Previous research has shown that fungal community diversity is positively correlated with plant diversity (Dassen et al., 2017). In the succession from grassland to bare land, pika activities caused the reduction of edible forages (Liu et al., 2013). These plant and humus changes may cause structural changes in the fungal community. In particular, most fungal communities became convergent. This suggested that only part of the taxa in the fungal community may remain in a bare land environment. This inadaptability of fungi in bald patches might allow other dominant species to inhabit different ecological niches.

Thirdly, the archaea communities thrived more in the old mound and showed an obvious differentiation. The unique adaptability of archaea in various environments has been confirmed (Bang and Schmitz, 2018). Members of microbial communities can colonize different habitats according to their nutritional preferences (Blagodatskaya and Kuzyakov, 2008). Archaea communities in sediments have been proved to have strong adaptability to serious hydrological interference (Gionchetta et al., 2019). Therefore, the community differentiation of archaea communities may also promote their colonization and reproduction. Importantly, the combined effects delayed this structural change compared with only pika activities. Therefore, we speculate that the combined effects of herbivores (i.e., pika and livestock) on the interaction or competition of food resources have a more positive influence on the microbiome than the pika disturbance alone.

### Multiple Environmental Factors Drive Microbial Communities

Among the measured parameters, pH had a very important impact on bacterial and archaeal communities, which is similar to the previous study (Fierer, 2017; Lammel et al., 2018). In our study, the soil became alkaline (8.2–8.5). We infer that this change could be as follows: (1) The high-intensity disturbance reduced plant biomass and increased the bare land area (Dong et al., 2013), theoretically reducing the buffering effect of plants on the change of soil pH due to external interference; (2) the returns of animal feces and urine may increase soil available nutrients and surface soil pH (Yu et al., 2017); (3) soil water evaporation might increase after the decrease of plant biomass, which may also lead to the increase of pH value. Based on our results, bacterial diversity decreased with the increase of pH, while the archaeal community showed the opposite trend. This decreased the abundance of those major bacterial taxa that were not adapted to the high pH environment. In contrast, more abundant and diverse archaeal taxa appeared in alkaline soils. This suggested that archaea may play a role in microbial communities under the mixed perturbation. In addition to pH, other environmental factors such as NH_4_-N, SOC, and TN were also significantly correlated with bacterial and archaeal communities (de Vries et al., 2012; Fierer, 2017; Wei et al., 2020). These factors affecting microbial communities are various rather than the individual. However, soil physicochemical properties had little effect on the habitat selection of the fungal community. This may be related to the selective effect of plant-fungal interaction (Dassen et al., 2017). In brief, the different strategies toward disturbance may drive different niche distributions of microbial communities to reduce their competition in the same habitat (Ghoul and Mitri, 2016; Wei et al., 2020).

### Soil Archaea Networks Are More Stable Than Bacterial and Fungal Networks Under Pika Disturbance

Network analysis was applied to explore the community co-occurrence pattern of community in response to disturbance. Firstly, regardless of the disturbance, the archaeal network had fewer links than bacterial and fungal networks. Some studies predicted that ecological networks composed of weak interactions are more stable than those composed of strong interactions (Neutel et al., 2002; Coyte Katharine et al., 2015). The complexity of microbial networks was also reduced by limited resources (Banerjee et al., 2019). This indicated that the archaea communities had simpler and more stable co-occurrence patterns than bacteria and fungi. In addition, the archaeal network possessed a higher average clustering coefficient. Studies have found that a larger network average clustering coefficient reflects a higher community functional redundancy (Sun et al., 2013; Li et al., 2017). This suggested that archaea communities might have high functional redundancy in responding to external disturbances, whereas bacterial and fungal networks appeared relatively fragile. Thirdly, the archaeal network was lower in the average degree and modularity. The lower modularity of a network is related to the stronger resilience of its community (Eldridge et al., 2015), and the degree of network has a positive correlation with the complexity of interspecific interactions (Wu et al., 2016). It means the simple archaeal networks may have a greater ability to cope with interferences than bacterial and fungal communities, thereby potentially moving toward another community balance.

Moreover, the negative correlations in the archaea network under the mixed disturbance were higher than that under the only pika disturbance. The existence of negative interactions indicates the increase in the potential competition within the community (Faust and Raes, 2012). This may stabilize the overall ecological networks (Coyte Katharine et al., 2015). Therefore, members of the archaea networks may compete and maintain the stability of networks in the disturbed environment. However, we should note that it is not sufficient to judge their roles or functions in the system only based on topological features or correlations (Faust, 2021). This is only a statistically determined association between the relative abundances of OTUs. The potential roles of microbial ecological networks remain to be further explored.

### Pika Disturbance Increases the Cross-Domain Cooperation Between Bacterial and Fungal Networks

Cross-domain networks were used to study the interaction among bacterial, fungal, and archaeal communities in response to interference. Consistent with our first hypothesis, the complexity and positive connections increased in the bacterial-fungal communities during the pika disturbance. It has been considered that the positively connected members in the community may make a series of responses to the external environmental interference and result in synergetic oscillation, increasing the community instability (Coyte Katharine et al., 2015). The cross-domain cooperation between bacteria and fungi was also reflected in the response to invasion by bacterial wilt pathogens (Tan et al., 2021). This may also explain the significant positive correlation between bacterial and fungal diversity (Figure 2A). The bacterial and fungal α diversity was significantly decreased, and they adopted another survival strategy, that is, to resist environmental interference by increasing cross-domain cooperation. Nevertheless, this also may increase network fragility between bacterial and fungal communities. In contrast, the archaea community had fewer links in the cross-domain networks. That is to say, the archaea in the microbial networks had a more independent position, which may contribute to the stability of the archaea community. Moreover, the joint disturbance of grazing and pika increased the cross-domain competition between bacteria and archaea. The possible competition of different taxa for resources has also been mentioned in the previous study (Banerjee et al., 2016). This cross-domain competition may contribute to the overall stability of the microbial community in response to disturbance.

### The Formation of New Mounds Reduces the Ecosystem Multifunctionality

The microbial community plays a key role in the ecosystem multifunctionality (EMF) (Bardgett and van der Putten, 2014; Wagg et al., 2014). This study suggested that EMF was positively correlated with bacterial diversity, but not with fungal and archaeal diversity. This was consistent with previous studies (Jing et al., 2015). Archaea, bacteria, and fungi differ in their ecological functions in soil ecosystems (Ma et al., 2017). Simultaneously, our results showed that the decrease in EMF was related to the increase in the negative correlations in the cross-domain microbial community networks. EMF decreased with the increase in the microbial community competition. This may also be related to the niche competitions between bacteria and archaea communities. In other words, the bacterial taxa originally undertaking certain functions may be reduced and replaced by some archaea, which may affect the EMF. Moreover, the mixed disturbance also played an important role in driving EMF. Vegetation diversity maintains the stability of EMF (Eisenhauer et al., 2018). As stated in the previous studies (Li et al., 2013; Sun et al., 2015; Eldridge et al., 2016) and our second hypothesis, there was little positive effect of high-intensity pika disturbance on EMF. Surprisingly, the combined disturbance did not exacerbate the reduction of EMF as significantly as the only pika interference in our study. Some researchers have also found that the interference of echidna on soil can to some extent counteract the negative impact of grazing on soil function (Eldridge et al., 2016). This suggests that the joint interference caused by herbivore coexistence may better offset the negative effects on EMF than we expected. Notably, we study the soil microbiomes and functions under the disturbance of pika with high population density, and the results of disturbance with different population densities may be different. Thus, more studies should focus on the impacts of the population densities of livestock and pika on the microbial community and EMF.

## Conclusion

This study revealed the responses of bacteria, fungi, archaea, and ecosystem multifunctionality to only pika interference. Bacterial and fungal networks were less stable than archaeal networks. Under the interference, bacteria and fungi cooperated across domains, while archaeal and bacterial communities showed more competition. Bacteria, fungi, and archaea showed different strategies for responding to external disturbances. This study provides us with a deeper understanding of the impact of animal activities on microbial communities on the QTP. High-density pika activities reduced soil nutrition and the ecosystem multifunctionality. Therefore, the disturbance of pika at high density has an adverse impact on the ecosystem. Nevertheless, the combined effects weakened such influences. These results provide insights into designing reasonable ecological management strategies for alpine grassland ecosystems. Future studies may more focus on the effects of pika population density and grazing intensity on soil microbial communities and ecosystem functions.

## Data Availability Statement

The datasets presented in this study can be found in online repositories. The names of the repository/repositories and accession number(s) can be found in the article/Supplementary Material.

## Author Contributions

HL, JQ, and XL designed this experiment and provide the funding. HL and JY finished the work of methodology, project administration, and resources. JY did the data analysis, manuscript writing, and editing. The manuscript was reviewed, and edited in close consultation with all authors.

## Conflict of Interest

YZ was employed by Changsha Central South Forestry Survey Planning and Design Co., Ltd. The remaining authors declare that the research was conducted in the absence of any commercial or financial relationships that could be construed as a potential conflict of interest. The reviewer TB declared a shared affiliation with the several authors JQ, XL, and HL at the time of the review.

## Publisher’s Note

All claims expressed in this article are solely those of the authors and do not necessarily represent those of their affiliated organizations, or those of the publisher, the editors and the reviewers. Any product that may be evaluated in this article, or claim that may be made by its manufacturer, is not guaranteed or endorsed by the publisher.

## Footnotes

1
https://rdp.cme.msu.edu
