# Supplementary material for: Animal Activities of the Key Herbivore Plateau Pika (Ochotona curzoniae) on the Qinghai-Tibetan Plateau Affect Grassland Microbial Networks and Ecosystem Functions
Source: Front Microbiol. 2022 Jul 6;13:950811. doi: 10.3389/fmicb.2022.950811 (PMC9298508; doi:10.3389/fmicb.2022.950811)
Supplement: Supplementary file 1 [file Data_Sheet_1.docx]

**Animal activities of the key herbivore plateau pika (*Ochotona curzoniae*) on the Qinghai-Tibetan Plateau affect grassland microbial networks and ecosystem functions**

Jiawei Yang^1^, Sijie Wang^1^, Wanghong Su^1^, Qiaoling Yu^1^, Xiaochen Wang^1^, Qian Han^1^, Yuting Zheng^6^, Jiapeng Qu^2,3^*, Xiangzhen Li^4^*, Huan Li^1, 2,5^*

1. School of Public Health, Lanzhou University, Lanzhou, 730000, China
2. Key Laboratory of Adaptation and Evolution of Plateau Biota, Northwest Institute of Plateau Biology, Chinese Academy of Sciences, Xining, Qinghai, 810008, China.
3. Qinghai Provincial Key Laboratory of Restoration Ecology for Cold Region, Northwest Institute of Plateau Biology, Chinese Academy of Sciences, Xining, 810008, China.
4. Key Laboratory of Environmental and Applied Microbiology, Environmental Microbiology Key Laboratory of Sichuan Province, Chengdu Institute of Biology, Chinese Academy of Sciences, Chengdu, 610041, China.
5. State Key Laboratory of Grassland Agro-ecosystems, Center for Grassland Microbiome, College of pastoral agriculture science and technology, Lanzhou University, Lanzhou, 730000, China.
6. Changsha Central South Forestry Survey Planning and Design Co. LTD, Changsha, 410014, China

^*^Corresponding author, Huan Li, *E-mail: lihuanzky@163.com; lixz@cib.ac.cn (X.L); *E-mail jpqu@nwipb.cas.cn (J.Q.).

**Supporting files:**

**Table S1-S11**

**Figure S1-S6**

**Table S1.** Two-way analysis of variance (Two-way ANOVA) showing the effects of disturbance type and surface type on the ecosystem multifunctionality (EMF) and environmental factors.

| Two-way ANOVA | Disturbance type | | Surface type | | Disturbance type *  Surface type | |
| --- | --- | --- | --- | --- | --- | --- |
|  | F | *P* | F | *P* | F | *P* |
| EMF | 10.085 | **<0.001***** | 23.576 | **<0.001***** | 0.554 | 0.697 |
|  |  |  |  |  |  |  |
| Dry Weight (g) | 13.096 | **<0.001***** | 221.925 | **<0.001***** | 0.400 | 0.808 |
| Wet Weight (g) | 8.260 | **<0.001***** | 62.895 | **<0.001***** | 2.936 | **0.024*** |
| Total Coverage (%) | 8.247 | **<0.001***** | 50.770 | **<0.001***** | 2.770 | **0.031*** |
|  |  |  |  |  |  |  |
| NO_3_-N (mg/kg) | 15.403 | **<0.001***** | 3.198 | **0.016*** | 2.656 | **0.037*** |
| pH | 10.180 | **<0.001***** | 16.990 | **<0.001***** | 18.919 | **<0.001***** |
| NH_4_-N (mg/kg) | 6.119 | **0.003**** | 0.546 | 0.702 | 6.286 | **<0.001***** |
| CON (mS/cm) | 5.343 | **0.006**** | 2.672 | **0.036*** | 8.375 | **<0.001***** |
| SOC (g/kg) | 4.767 | **0.011*** | 2.428 | 0.053 | 2.901 | **0.026*** |
| TN (g/kg) | 3.734 | **0.027*** | 2.401 | 0.055 | 2.881 | **0.026*** |

Abbreviations: CON, conductivity; NH_4_-N, ammonium nitrogen; NO_3_-N, nitrate-nitrogen; SOC, soil organic carbon; TN, total nitrogen; Significant indicator by **P* < 0.05, ***P* < 0.01, and *** *P* <0.001.

**Table S2.** Two-way analysis of variance (Two-way ANOVA) showing the effects of disturbance type and surface type on bacterial, fungal, archaeal communities at the phylum level.

| Two-way ANOVA | Disturbance type | | Surface type | | Disturbance type * Surface type | |
| --- | --- | --- | --- | --- | --- | --- |
|  | F | *P* | F | *P* | F | *P* |
| **Bacteria** |  |  |  |  |  |  |
| Proteobacteria | 14.774 | **<0.001***** | 6.843 | **<0.001***** | 1.177 | 0.326 |
| Actinobacteria | 1.960 | 0.146 | 0.369 | 0.830 | 0.874 | 0.482 |
| Bacteroidetes | 2.387 | 0.097 | 10.231 | **<0.001***** | 2.569 | **0.043*** |
| Acidobacteria | 0.528 | 0.591 | 1.721 | 0.151 | 2.281 | 0.066 |
| Chloroflexi | 3.043 | 0.052 | 10.984 | **<0.001***** | 1.795 | 0.136 |
| Planctomycetes | 4.617 | **0.012*** | 3.132 | **0.018*** | 3.308 | **0.014*** |
| Gemmatimonadetes | 3.540 | **0.033*** | 9.560 | **<0.001***** | 1.268 | 0.288 |
| Firmicutes | 2.116 | 0.126 | 0.841 | 0.502 | 3.614 | **0.009**** |
| Nitrospirae | 3.342 | **0.039*** | 2.497 | **0.047*** | 1.945 | 0.109 |
| Spirochaetes | 4.108 | **0.019*** | 0.130 | 0.971 | 0.244 | 0.913 |
| **Fungi** |  |  |  |  |  |  |
| Ascomycota | 2.144 | 0.123 | 0.576 | 0.681 | 0.625 | 0.646 |
| Basidiomycota | 0.532 | 0.589 | 0.873 | 0.483 | 0.718 | 0.582 |
| Rozellomycota | 0.570 | 0.567 | 4.080 | **0.004**** | 2.114 | 0.085 |
| Glomeromycota | 3.993 | **0.021*** | 2.946 | **0.024*** | 1.649 | 0.168 |
| Incertae sedis | 7.785 | **0.001**** | 0.375 | 0.826 | 1.444 | 0.225 |
| Zygomycota | 41.016 | **<0.001***** | 0.076 | 0.989 | 0.085 | 0.987 |
| Chytridiomycota | 64.029 | **<0.001***** | 0.003 | 1.000 | 0.004 | 1.000 |
| **Archaea** |  |  |  |  |  |  |
| Crenarchaeota | 2.067 | 0.132 | 4.960 | **0.001**** | 1.187 | 0.321 |
| Euryarchaeota | 1.782 | 0.174 | 4.643 | **0.002**** | 1.056 | 0.382 |
| Parvarchaeota | 3.095 | 0.050 | 4.192 | **0.004**** | 2.232 | 0.071 |

Abbreviations: Significant indicator by **P* < 0.05, ***P* < 0.01, and *** *P* <0.001.

**Table S3.** Two-way analysis of variance (Two-way ANOVA) showing the effects of disturbance type and surface type on bacterial, fungal, archaeal communities at the genus level.

| Two-way ANOVA | Disturbance type | | Surface type | | Disturbance type * Surface type | |
| --- | --- | --- | --- | --- | --- | --- |
|  | F | *P* | F | *P* | F | *P* |
| **Bacteria** |  |  |  |  |  |  |
| Solirubrobacterales(UG) | 2.191 | 0.117 | 4.762 | **0.001**** | 0.432 | 0.785 |
| Rhodospirillales(UG) | 11.129 | **<0.001***** | 3.672 | **0.008**** | 0.817 | 0.517 |
| RB41(UG) | 3.910 | **0.023*** | 4.405 | **0.003**** | 2.115 | 0.085 |
| Cytophagaceae(UG) | 6.840 | **0.002**** | 1.520 | 0.202 | 2.265 | 0.067 |
| Chitinophagaceae(UG) | 8.402 | **<0.001***** | 6.025 | **<0.001***** | 1.504 | 0.207 |
| Sinobacteraceae(UG) | 14.224 | **<0.001***** | 3.797 | **0.006**** | 2.364 | 0.058 |
| iii1-15(UG) | 4.803 | **0.010*** | 3.057 | **0.020*** | 3.782 | **0.007**** |
| Gaiellaceae(UG) | 9.020 | **<0.001***** | 1.465 | 0.219 | 0.707 | 0.589 |
| Rhodospirillaceae(UG) | 5.115 | **0.008**** | 2.091 | 0.088 | 0.989 | 0.417 |
| Ellin6529(UG) | 18.185 | **<0.001***** | 4.556 | **0.002**** | 0.817 | 0.517 |
| **Fungi** |  |  |  |  |  |  |
| Eurotiales(UG) | 6.114 | **0.003**** | 0.279 | 0.891 | 0.349 | 0.844 |
| Ascomycota(UG) | 0.134 | 0.875 | 3.581 | **0.009**** | 1.293 | 0.278 |
| Stephanosporaceae(UG) | 0.398 | 0.673 | 0.346 | 0.846 | 1.333 | 0.263 |
| *Gymnoascus* | 0.767 | 0.467 | 1.295 | 0.277 | 1.807 | 0.133 |
| *Phialemonium* | 0.526 | 0.592 | 0.757 | 0.556 | 0.722 | 0.579 |
| Microascales(UG) | 0.718 | 0.490 | 1.254 | 0.293 | 1.123 | 0.350 |
| *Exophiala* | 0.880 | 0.418 | 1.182 | 0.323 | 1.120 | 0.351 |
| Chaetothyriales(UG) | 5.443 | **0.006**** | 0.489 | 0.744 | 1.257 | 0.292 |
| *Mortierella* | 11.663 | **<0.001***** | 0.654 | 0.625 | 0.396 | 0.811 |
| *Podospora* | 1.489 | 0.231 | 2.035 | 0.095 | 1.812 | 0.132 |
| **Archaea** |  |  |  |  |  |  |
| *Candidatus Nitrososphaera* | 2.037 | 0.136 | 4.941 | **0.001**** | 1.157 | 0.335 |
| *Methanobrevibacter* | 0.155 | 0.856 | 3.412 | **0.012*** | 3.233 | **0.015*** |
| YLA114(UG) | 3.204 | **0.045*** | 4.129 | **0.004*** | 2.312 | 0.063 |
| E2(UG) | 1.170 | 0.315 | 6.000 | **<0.001***** | 2.269 | 0.067 |
| *Halorubrum* | 0.009 | 0.991 | 2.561 | **0.043*** | 0.715 | 0.583 |
| *WCHD3-30* | 0.190 | 0.827 | 4.102 | **0.004**** | 0.401 | 0.808 |
| *Nitrosopumilus* | 0.013 | 0.987 | 1.248 | 0.296 | 1.981 | 0.103 |
| *Methanosphaera* | 0.839 | 0.435 | 0.773 | 0.545 | 0.238 | 0.917 |
| *Halolamina* | 1.310 | 0.275 | 1.021 | 0.401 | 1.158 | 0.334 |
| Micrarchaeles(UG) | 1.006 | 0.369 | 1.061 | 0.380 | 0.401 | 0.808 |

Abbreviations: UG, unidentified genus; Significant indicator by **P* < 0.05, ***P* < 0.01, and *** *P* <0.001.

**Table S4.** Mann-Whitney *U*-Test showing the differences in the alpha diversity of bacteria, fungi, archaea between groups.

| M-W *U* Test | Bacteria |  | Fungi |  | Archaea |
| --- | --- | --- | --- | --- | --- |
|  | *P* |  | *P* |  | *P* |
| PG vs. C | 0.631 |  | 0.393 |  | 0.165 |
| PLG vs. C | 0.075 |  | 0.063 |  | 0.165 |
|  |  |  |  |  |  |
| PG vs. PN | **0.002**** |  | 0.631 |  | 0.165 |
| PN vs. PO | 0.190 |  | 0.353 |  | 0.631 |
| PO vs. PB | 0.739 |  | 0.436 |  | 0.19 |
|  |  |  |  |  |  |
| PLG vs. PLN | **0.029*** |  | 0.165 |  | 0.529 |
| PLN vs. PLO | 0.353 |  | 0.796 |  | 0.436 |
| PLO vs. PLB | 0.063 |  | **0.029*** |  | 0.315 |
|  |  |  |  |  |  |
| PG vs. PLG | 0.315 |  | 0.063 |  | 0.853 |
| PN vs. PLN | **0.003**** |  | 0.218 |  | **0.005**** |
| PO vs. PLO | 0.739 |  | 0.315 |  | **0.029*** |
| PB vs. PLB | 0.052 |  | 0.218 |  | **0.043*** |
| PI vs. PLI | 0.684 |  | 1.000 |  | 0.075 |
|  |  |  |  |  |  |
| PI vs. PG | 0.796 |  | 0.247 |  | 0.796 |
| PLI vs. PLG | 0.190 |  | 0.436 |  | 0.165 |

Abbreviations: C, natural grass; P, pika disturbance; PL, grazing and pika disturbance; G, grassland; N, new mound; O, old mound; B, bare land; I, inside the mound. Significant indicator by **P* < 0.05, ***P* < 0.01, and *** *P* <0.001.

**Table S5.** Permutational multivariate analysis of variance (PERMANOVA) analysis showing the comparison between different groups based on Bray-Curtis distance metrics.

| PERMANOVA | Bacteria | | | | | |  | | Fungi | | | | | |  | | Archaea | | | | | |  |
| --- | --- | --- | --- | --- | --- | --- | --- | --- | --- | --- | --- | --- | --- | --- | --- | --- | --- | --- | --- | --- | --- | --- | --- |
|  | F | | *R^2^* | | *P* | |  | | F | | *R^2^* | | *P* | |  | | F | | *R^2^* | | *P* | |  |
| PG vs. C | | 5.590 | | 0.237 | | **0.001***** | |  | | 2.280 | | 0.112 | | 0.066 | |  | | 6.577 | | 0.268 | | **0.003**** | |
| PLG vs. C | | 10.663 | | 0.372 | | **0.001***** | |  | | 1.986 | | 0.099 | | 0.077 | |  | | 16.029 | | 0.471 | | **0.001***** | |
|  | |  | |  | |  | |  | |  | |  | |  | |  | |  | |  | |  | |
| PG vs. PN | | 3.344 | | 0.157 | | **0.001***** | |  | | 1.659 | | 0.084 | | 0.106 | |  | | 1.040 | | 0.055 | | 0.359 | |
| PN vs. PO | | 1.366 | | 0.071 | | 0.090 | |  | | 0.832 | | 0.044 | | 0.568 | |  | | 1.564 | | 0.080 | | 0.209 | |
| PO vs. PB | | 1.711 | | 0.087 | | 0.053 | |  | | 1.325 | | 0.069 | | 0.194 | |  | | 2.465 | | 0.120 | | 0.051 | |
|  | |  | |  | |  | |  | |  | |  | |  | |  | |  | |  | |  | |
| PLG vs. PLN | | 2.528 | | 0.123 | | **0.001***** | |  | | 1.471 | | 0.076 | | 0.120 | |  | | 2.968 | | 0.142 | | **0.017*** | |
| PLN vs. PLO | | 0.991 | | 0.052 | | 0.394 | |  | | 1.247 | | 0.065 | | 0.221 | |  | | 1.194 | | 0.062 | | 0.293 | |
| PLO vs. PLB | | 1.038 | | 0.055 | | 0.349 | |  | | 1.039 | | 0.055 | | 0.384 | |  | | 2.465 | | 0.120 | | 0.054 | |
|  | |  | |  | |  | |  | |  | |  | |  | |  | |  | |  | |  | |
| PG vs. PLG | | 1.593 | | 0.081 | | **0.018*** | |  | | 1.260 | | 0.065 | | 0.222 | |  | | 1.657 | | 0.084 | | 0.166 | |
| PN vs. PLN | | 1.473 | | 0.076 | | **0.036*** | |  | | 0.996 | | 0.052 | | 0.417 | |  | | 1.564 | | 0.080 | | 0.202 | |
| PO vs. PLO | | 1.168 | | 0.061 | | 0.205 | |  | | 1.672 | | 0.085 | | 0.081 | |  | | 5.376 | | 0.230 | | **0.001***** | |
| PB vs. PLB | | 1.321 | | 0.068 | | 0.142 | |  | | 1.941 | | 0.097 | | 0.088 | |  | | 0.846 | | 0.045 | | 0.514 | |
| PI vs. PLI | | 1.323 | | 0.068 | | 0.114 | |  | | 1.348 | | 0.070 | | 0.196 | |  | | 2.297 | | 0.113 | | 0.064 | |
|  | |  | |  | |  | |  | |  | |  | |  | |  | |  | |  | |  | |
| PI vs. PG | | 2.006 | | 0.100 | | **0.001***** | |  | | 1.844 | | 0.093 | | 0.088 | |  | | 1.243 | | 0.065 | | 0.250 | |
| PLI vs. PLG | | 2.480 | | 0.121 | | **0.001***** | |  | | 2.072 | | 0.103 | | 0.053 | |  | | 1.800 | | 0.091 | | 0.127 | |

Abbreviations: C, natural grass; P, pika disturbance; PL, grazing and pika disturbance; G, grassland; N, new mound; O, old mound; B, bare land; I, inside the mound. Significant indicator by **P* < 0.05, ***P* < 0.01, and *** *P* <0.001.

**Table S6.** Multiple regression matrix (MRM) analysis showing the effect of these three factors on the bacterial, fungal, and archaeal communities based on Bray-Curtis distance metrics.

| MRM | Bacteria | |  | Fungi | |  | Archaea | |
| --- | --- | --- | --- | --- | --- | --- | --- | --- |
|  | *R^2^* | *P* |  | *R^2^* | *P* |  | *R^2^* | *P* |
| Combined disturbance | 0.039 | **0.001***** |  | 0.002 | **0.019*** |  | 0.023 | **0.001***** |
| Pika disturbance | 0.006 | **0.009**** |  | <0.001 | 0.074 |  | 0.002 | **0.028*** |
| Environmental factors | 0.024 | **0.001***** |  | 0.002 | **0.039*** |  | <0.001 | 0.926 |

Abbreviations: Significant indicator by **P* < 0.05, ***P* < 0.01, and *** *P* <0.001.

**Table S7.** Permutational multivariate analysis of variance (PERMANOVA) analysis showing the effects of driving factors on the bacterial, fungal, and archaeal communities based on Bray-Curtis distance metrics.

| PERMANOVA | Bacteria | | |  | | Fungi | | | |  | | Archaea | | |
| --- | --- | --- | --- | --- | --- | --- | --- | --- | --- | --- | --- | --- | --- | --- |
|  | F | *R^2^* | *P* | |  | | F | *R^2^* | *P* |  | F | | *R^2^* | *P* |
| Combined disturbance | 4.358 | 0.035 | **0.001***** | |  | | 2.515 | 0.022 | **0.022*** |  | 6.654 | | 0.052 | **0.001***** |
| pH | 4.242 | 0.034 | **0.001***** | |  | | 1.489 | 0.013 | 0.134 |  | 3.400 | | 0.027 | **0.029*** |
| Pika disturbance | 3.842 | 0.031 | **0.001***** | |  | | 2.723 | 0.023 | **0.012*** |  | 3.472 | | 0.027 | **0.015*** |
| Total Coverage(%) | 2.554 | 0.206 | **0.001***** | |  | | 0.730 | 0.006 | 0.709 |  | 3.238 | | 0.025 | **0.019*** |
| TN(g/kg) | 2.355 | 0.019 | **0.002**** | |  | | 2.270 | 0.020 | **0.023*** |  | 1.228 | | 0.010 | 0.285 |
| CON(mS/cm) | 1.809 | 0.015 | **0.017*** | |  | | 1.505 | 0.130 | 0.112 |  | 1.696 | | 0.013 | 0.125 |
| SOC(mg/kg) | 1.688 | 0.014 | **0.020*** | |  | | 1.580 | 0.014 | 0.123 |  | 3.102 | | 0.024 | **0.016*** |
| NO_3_-N(mg/kg) | 1.673 | 0.013 | **0.023*** | |  | | 1.557 | 0.010 | 0.248 |  | 2.273 | | 0.018 | 0.062 |
| NH_4_-N(g/kg) | 1.491 | 0.012 | 0.051 | |  | | 0.489 | 0.004 | 0.928 |  | 2.093 | | 0.016 | 0.082 |
| Wet Weight(g) | 1.366 | 0.011 | 0.099 | |  | | 1.066 | 0.009 | 0.357 |  | 1.035 | | 0.008 | 0.352 |
| Dry Weight(g) | 0.931 | 0.007 | 0.544 | |  | | 2.617 | 0.023 | **0.016*** |  | 1.812 | | 0.014 | 0.127 |

Abbreviations: CON, conductivity; NH_4_-N, ammonium nitrogen; NO_3_-N, nitrate-nitrogen; SOC, soil organic carbon; TN, total nitrogen; Significant indicator by **P* < 0.05, ***P* < 0.01, and *** *P* <0.001.

**Table S8.** The detailed properties of bacterial, fungal, and archaeal networks of Fig. 5.

Abbreviations: C, natural grass; P, pika disturbance; PL, grazing and pika disturbance; G, grassland; N, new mound; O, old mound; B, bare land; I, inside the mound.

|  | Group | Node | Edge | Average degree | Average  path length | Average  clustering  coefficient | Modularity | Diameter | Density | Positive  (%) | Negative  (%) |
| --- | --- | --- | --- | --- | --- | --- | --- | --- | --- | --- | --- |
| Bacteria | PG | 147 | 760 | 10.34 | 2.934 | 0.423 | 8.205 | 6 | 0.071 | 52.11 | 47.89 |
|  | PN | 154 | 1043 | 13.545 | 2.79 | 0.474 | 1.135 | 7 | 0.089 | 70.18 | 29.82 |
|  | PO | 155 | 1042 | 13.445 | 2.89 | 0.445 | 3.163 | 9 | 0.087 | 56.91 | 43.09 |
|  | PB | 154 | 2073 | 26.922 | 2.491 | 0.583 | 0.498 | 8 | 0.176 | 75.3 | 24.7 |
|  | PLG | 148 | 865 | 11.689 | 2.959 | 0.434 | 0.939 | 8 | 0.08 | 71.79 | 28.21 |
|  | PLN | 151 | 826 | 10.94 | 3.07 | 0.424 | 3.38 | 8 | 0.073 | 57.26 | 42.74 |
|  | PLO | 154 | 1128 | 14.649 | 2.927 | 0.491 | 1.702 | 7 | 0.096 | 64.63 | 35.37 |
|  | PLB | 149 | 1703 | 22.859 | 2.418 | 0.552 | 1.242 | 7 | 0.154 | 60.78 | 39.22 |
|  |  |  |  |  |  |  |  |  |  |  |  |
| Fungi | PG | 169 | 2619 | 30.994 | 2.389 | 0.609 | 0.498 | 8 | 0.184 | 87.29 | 12.71 |
|  | PN | 181 | 1388 | 15.337 | 2.903 | 0.445 | 0.976 | 7 | 0.085 | 75.65 | 24.35 |
|  | PO | 182 | 2811 | 30.89 | 2.865 | 0.649 | 1.659 | 9 | 0.171 | 61.72 | 38.28 |
|  | PB | 180 | 4680 | 52 | 2.239 | 0.653 | 0.998 | 8 | 0.291 | 66.28 | 33.72 |
|  | PLG | 182 | 3074 | 33.78 | 2.355 | 0.62 | 0.595 | 6 | 0.187 | 81.88 | 18.12 |
|  | PLN | 182 | 1218 | 13.385 | 3.016 | 0.437 | 0.954 | 6 | 0.074 | 75.12 | 24.88 |
|  | PLO | 177 | 2222 | 25.107 | 2.609 | 0.532 | 0.598 | 9 | 0.143 | 83.93 | 16.07 |
|  | PLB | 174 | 1253 | 14.402 | 3.08 | 0.487 | 0.952 | 8 | 0.083 | 76.06 | 23.94 |
|  |  |  |  |  |  |  |  |  |  |  |  |
| Archaea | PG | 32 | 270 | 16.875 | 1.58 | 0.846 | 0.067 | 6 | 0.544 | 87.78 | 12.22 |
|  | PN | 36 | 197 | 10.944 | 2.081 | 0.818 | 0.351 | 6 | 0.313 | 81.22 | 17.88 |
|  | PO | 34 | 91 | 5.353 | 3.652 | 0.63 | 0.805 | 8 | 0.162 | 78.02 | 21.98 |
|  | PB | 38 | 213 | 11.211 | 2.239 | 0.646 | 0.277 | 5 | 0.303 | 87.32 | 12.68 |
|  | PLG | 33 | 92 | 5.576 | 2.039 | 0.56 | 1.122 | 6 | 0.174 | 66.3 | 33.7 |
|  | PLN | 38 | 60 | 4.737 | 2.898 | 0.668 | 0.771 | 7 | 0.128 | 82.22 | 17.88 |
|  | PLO | 39 | 151 | 7.744 | 2.183 | 0.594 | 0.872 | 5 | 0.204 | 76.82 | 23.18 |
|  | PLB | 39 | 116 | 5.949 | 2.725 | 0.61 | 0.987 | 8 | 0.157 | 70.69 | 29.31 |

**Table S9.** The detailed properties of the microbial (bacteria, fungi, and archaea) networks of Fig. 6.

| Group | Node | Edge | Average degree | Average path length | Average clustering coefficient | Modularity | Diameter | Density | Positive(%) | Negative(%) |
| --- | --- | --- | --- | --- | --- | --- | --- | --- | --- | --- |
| PG | 356 | 2166 | 12.169 | 3.426 | 0.093 | 0.488 | 9 | 0.034 | 69.58 | 30.42 |
| PN | 361 | 1753 | 9.712 | 3.679 | 0.075 | 0.478 | 8 | 0.027 | 63.89 | 36.11 |
| PO | 369 | 2337 | 12.667 | 3.154 | 0.130 | 0.480 | 8 | 0.034 | 52.67 | 47.33 |
| PB | 359 | 2557 | 14.245 | 3.221 | 0.080 | 0.396 | 9 | 0.040 | 57.96 | 42.04 |
| PLG | 356 | 2405 | 13.511 | 3.463 | 0.135 | 0.374 | 9 | 0.038 | 73.26 | 26.74 |
| PLN | 359 | 1984 | 11.053 | 3.401 | 0.128 | 0.496 | 8 | 0.031 | 50.00 | 50.00 |
| PLO | 351 | 3242 | 18.473 | 3.071 | 0.147 | 0.340 | 8 | 0.053 | 60.86 | 39.14 |
| PLB | 343 | 2152 | 12.548 | 3.213 | 0.163 | 0.442 | 8 | 0.037 | 51.30 | 48.70 |

Abbreviations: C, natural grass; P, pika disturbance; PL, grazing and pika disturbance; G, grassland; N, new mound; O, old mound; B, bare land; I, inside the mound. Significant indicator by **P* < 0.05, ***P* < 0.01, and *** *P* <0.001.

**Table S10.** The connection, positive, and negative proportion of bacteria, fungi, and archaea to each other in the network of Fig.6.

|  | Group | Connection(%) | | | |  | Positive(%) | | | |  | Negative(%) | | | |
| --- | --- | --- | --- | --- | --- | --- | --- | --- | --- | --- | --- | --- | --- | --- | --- |
|  |  | G | N | O | B |  | G | N | O | B |  | G | N | O | B |
| Bacteria-Fungi | P | 1.94 | 0.46 | 2.40 | 6.26 |  | 1.89 | 0.61 | 2.88 | 6.78 |  | 1.78 | 0.16 | 1.67 | 4.36 |
|  | PL | 2.08 | 0.76 | 3.76 | 1.21 |  | 2.68 | 0.50 | 5.27 | 1.33 |  | 0.15 | 0.99 | 1.97 | 1.02 |
|  |  |  |  |  |  |  |  |  |  |  |  |  |  |  |  |
| Bacteria-Archaea | P | 0.69 | 1.14 | 0.68 | 1.64 |  | 0.76 | 1.57 | 0.70 | 1.42 |  | 0.44 | 0.31 | 0.62 | 1.66 |
|  | PL | 0.33 | 0.50 | 1.82 | 1.25 |  | 0.22 | 0.60 | 1.78 | 0.80 |  | 0.61 | 0.40 | 1.64 | 1.67 |
|  |  |  |  |  |  |  |  |  |  |  |  |  |  |  |  |
| Fungi-Bacteria | P | 58.82 | 50.26 | 65.98 | 79.55 |  | 49.84 | 47.65 | 66.17 | 75.52 |  | 71.30 | 52.52 | 61.06 | 70.53 |
|  | PL | 67.57 | 63.96 | 65.82 | 51.72 |  | 69.38 | 61.42 | 50.34 | 57.47 |  | 54.27 | 64.78 | 71.75 | 42.83 |
|  |  |  |  |  |  |  |  |  |  |  |  |  |  |  |  |
| Fungi-Archaea | P | 1.94 | 0.34 | 0.39 | 0.39 |  | 2.52 | 0.52 | 0.47 | 0.43 |  | 0.30 | 0.00 | 0.26 | 0.26 |
|  | PL | 0.91 | 0.10 | 1.17 | 0.33 |  | 0.77 | 0.00 | 2.15 | 0.27 |  | 1.22 | 0.20 | 0.16 | 0.37 |
|  |  |  |  |  |  |  |  |  |  |  |  |  |  |  |  |
| Archaea-Bacteria | P | 15.42 | 39.25 | 18.70 | 14.00 |  | 13.78 | 37.20 | 15.20 | 10.91 |  | 17.01 | 41.04 | 21.32 | 15.78 |
|  | PL | 10.35 | 21.82 | 17.49 | 32.62 |  | 9.13 | 22.53 | 17.84 | 24.05 |  | 12.50 | 20.54 | 15.08 | 39.78 |
|  |  |  |  |  |  |  |  |  |  |  |  |  |  |  |  |
| Archaea-Fungi | P | 25.76 | 10.33 | 15.32 | 6.45 |  | 31.21 | 12.46 | 14.58 | 4.93 |  | 9.17 | 5.97 | 15.07 | 7.41 |
|  | PL | 22.08 | 14.21 | 16.69 | 15.66 |  | 17.82 | 14.96 | 22.62 | 16.09 |  | 31.25 | 13.10 | 9.40 | 14.34 |

Abbreviations: P, pika disturbance; PL, grazing and pika disturbance; G, grassland; N, new mound; O, old mound; B, bare land.

**Table S11.** Mantel test showing the effects of driving factors on the bacterial, fungal, and archaeal communities based on Bray-Curtis distance metrics.

| Mantel test | Bacteria | |  | Fungi | |  | Archaea | |
| --- | --- | --- | --- | --- | --- | --- | --- | --- |
|  | *r* | *P* |  | *r* | *P* |  | *r* | *P* |
| Grazing | 0.232 | **0.001***** |  | 0.131 | **0.001***** |  | 0.192 | **0.001***** |
| pH | 0.300 | **0.001***** |  | 0.110 | **0.004**** |  | 0.210 | **0.001***** |
| Disturbance | 0.085 | **0.002**** |  | 0.058 | **0.019*** |  | 0.068 | **0.008**** |
| Total Coverage(%) | 0.092 | 0.051 |  | 0.092 | **0.013*** |  | 0.107 | **0.012*** |
| TN(g/kg) | 0.200 | **0.001***** |  | -0.025 | 0.771 |  | 0.060 | 0.060 |
| CON(mS/cm) | 0.069 | 0.133 |  | 0.057 | 0.077 |  | -0.001 | 0.497 |
| SOC(mg/kg) | 0.009 | 0.324 |  | 0.024 | 0.097 |  | 0.001 | 0.468 |
| NO_3_-N(mg/kg) | 0.118 | **0.001***** |  | 0.029 | 0.134 |  | 0.054 | **0.038*** |
| NH_4_-N(g/kg) | 0.198 | **0.001***** |  | -0.019 | 0.704 |  | 0.065 | 0.050 |
| Wet Weight(g) | 0.099 | 0.071 |  | 0.090 | **0.030*** |  | 0.109 | **0.021*** |
| Dry Weight(g) | 0.111 | **0.030*** |  | 0.082 | **0.039*** |  | 0.117 | **0.014*** |

Abbreviations: CON, conductivity; NH_4_-N, ammonium nitrogen; NO_3_-N, nitrate-nitrogen; SOC, soil organic carbon; TN, total nitrogen; Significant indicator by **P* < 0.05, ***P* < 0.01, and *** *P* <0.001.





**I**

**H**

**G**

**F**

**E**

**D**

**C**

**B**

**A**

**Fig. S1.** The changes of total coverage (A), wet weight (B), dry weight (C), pH (D), CON (E, conductivity), NO_3_-N (F, nitrate-nitrogen), NH_4_-N (G, ammonium nitrogen), TN (H, total nitrogen), and SOC (I, soil organic carbon) between different groups. C (orange), natural grass; P (blue), pika disturbance; PL (pink), grazing and pika disturbance; G, grassland; N, new mound; O, old mound; B, bare land; I, inside the mound. Significant indicator by **P* < 0.05, ***P* < 0.01, and *** *P* <0.001.


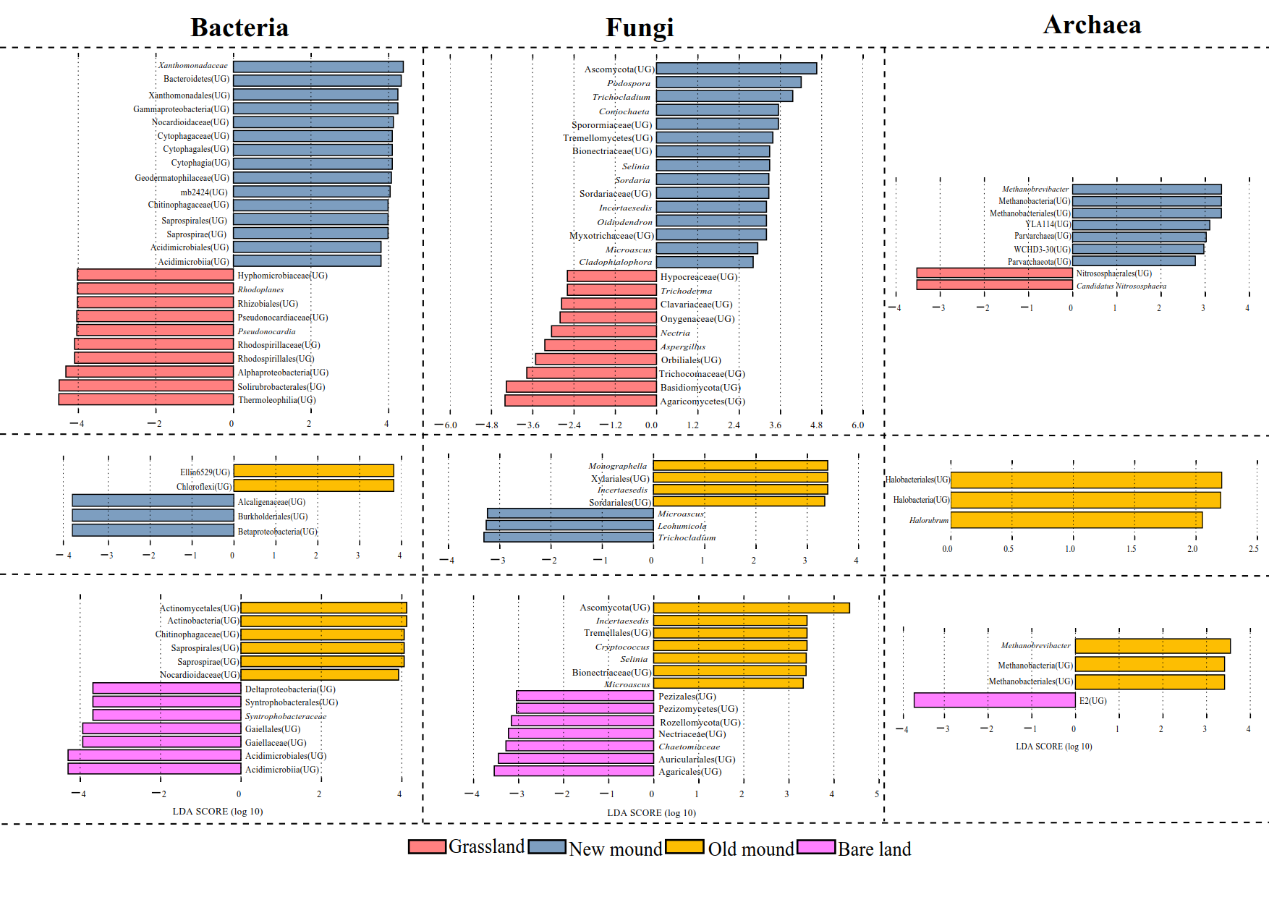


**Fig. S2.** The Linear discriminant analysis effect size (LEfSe) of bacterial (A), fungal (B), and archaeal (C) communities at the genus level between the different groups. UG, unidentified genus.

**C**

**B**

**A**





**C**

**B**

**A**

**Fig. S3.** The chao 1 index of bacterial (A), fungal (B), and archaeal (C) communities in the different groups. C, natural grass; P, pika disturbance; PL, grazing and pika disturbance; G, grassland; N, new mound; O, old mound; B, bare land; I, inside the mound. Significant indicator by **P* < 0.05, and ***P* < 0.01.

**C**

**B**

**A**





**Fig. S4.** Principal coordinate analysis (PCoA) analysis of bacterial (A, D), fungal (B, E), and archaeal (C, F) communities in the different groups based on Bray-Curtis distance metrics. The trajectory of beta diversity of bacterial (G), fungal (H), and archaeal (I) communities. C, natural grass; P, pika disturbance; PL, grazing and pika disturbance; G, grassland; N, new mound; O, old mound; B, bare land; I, inside the mound.

**I**

**H**

**G**

**F**

**E**

**D**





**Fig. S5.** Density, and proportion of negative correlation of bacterial (A), fungal (B), and archaeal (C) networks in Fig. 5. P (blue), pika disturbance; PL (pink), grazing and pika disturbance.

**A**

**C**

**B**















**J**

**I**

**H**

**G**

**F**

**E**

**D**

**C**

**A**

**B**

**Fig. S6.** The irregular correlation between fungal and archaeal α diversity (A), archaeal and bacterial α diversity (B), ecosystem multifunctionality (EMF) and fungal α diversity (C), and EMF and archaeal α diversity (D). The relationship between EMF and topological features of networks in Fig. 6 (E, average path length, F, diameter, G, average degree, H, average clustering coefficient, I, modularity, J, density). The area above and below the fitting line represents the 95% confidence interval.
